# Clinical experience with ipilimumab 3 mg/kg: real-world efficacy and safety data from an expanded access programme cohort

**DOI:** 10.1186/1479-5876-12-116

**Published:** 2014-05-07

**Authors:** Paolo A Ascierto, Ester Simeone, Vanna Chiarion Sileni, Jacopo Pigozzo, Michele Maio, Maresa Altomonte, Michele Del Vecchio, Lorenza Di Guardo, Paolo Marchetti, Ruggero Ridolfi, Francesco Cognetti, Alessandro Testori, Maria Grazia Bernengo, Michele Guida, Riccardo Marconcini, Mario Mandalà, Carolina Cimminiello, Gaetana Rinaldi, Massimo Aglietta, Paola Queirolo

**Affiliations:** 1 Istituto Nazionale Tumori Fondazione ‘G. Pascale’, Napoli, Italy; 2 Veneto Institute of Oncology IOV-IRCCS, Padua, Italy; 3 Istituto Toscano Tumori, University Hospital of Siena, Siena, Italy; 4 National Cancer Institute, Milan, Italy; 5 Dermopathic Institute of the Immaculate IDI-IRCCS, Rome, Italy; 6 Sant’ Andrea Hospital, University Sapienza, Rome, Italy; 7 Romagna National Cancer Institute, Meldola, Italy; 8 Regina Elena National Cancer Institute, Rome, Italy; 9 Divisione Melanoma e Sarcomi Muscolo-Cutanei, Istituto Europeo di Oncologia, Milan, Italy; 10 University Hospital St John the Baptist, Turin, Italy; 11 National Cancer Research Center, ‘Giovanni Paolo II’, Bari, Italy; 12 University Hospital Pisa, ‘Gathered Hospitals of Santa Chiara’, Pisa, Italy; 13 ‘Papa Giovanni XXIII’ Hospital, Bergamo, Italy; 14 San Raffaele Hospital, Milan, Italy; 15 ‘Paolo Giaccone’ Polyclinic University Hospital, Palermo, Italy; 16 Institute of Cancer Research and Treatment, Piedmont Oncology Foundation, Candiolo, Italy; 17 National Institute for Cancer Research, San Martino Hospital, Genoa, Italy; 18 Unit of Melanoma, Cancer Immunotherapy and Innovative Therapy Unit, Istituto Nazionale Tumori Fondazione ‘G. Pascale’ Via Mariano Semmola, 80131 Napoli, Italy

**Keywords:** Efficacy, Expanded access programme, Ipilimumab, Melanoma, Safety

## Abstract

**Background:**

Ipilimumab improves survival in patients with advanced melanoma. The activity and safety of ipilimumab outside of a clinical trial was assessed in an expanded access programme (EAP).

**Methods:**

Ipilimumab was available upon physician request for patients aged 16 or over with pretreated stage III (unresectable)/IV melanoma, for whom no other therapeutic option was available. Patients received ipilimumab 3 mg/kg every 3 weeks for four doses. Patients with stable disease or an objective response to ipilimumab were eligible for retreatment upon disease progression. Tumour assessments were conducted at baseline and week 12. Patients were monitored for adverse events (AEs) within 3 to 4 days of each scheduled visit.

**Results:**

Of 855 patients participating in the EAP in Italy, 833 were evaluable for response. Of these, 13% had an objective immune response, and the immune-related disease control rate was 34%. Median progression-free survival and overall survival were 3.7 and 7.2 months, respectively. Efficacy was independent of BRAF and NRAS mutational status. Overall, 33% of patients reported an immune-related AE (irAE). The frequency of irAEs was not associated with response to ipilimumab.

**Conclusions:**

Outside of a clinical trial setting, ipilimumab is a feasible treatment option in patients with pretreated metastatic melanoma, regardless of BRAF and NRAS mutational status. Data from this large cohort of patients support clinical trial evidence that ipilimumab can induce durable disease control and long-term survival in patients who have failed to respond to prior treatment.

## Introduction

Ipilimumab is a monoclonal antibody that blocks cytotoxic T-lymphocyte-associated antigen-4 (CTLA-4), a negative regulator of T-cell activation, thereby augmenting proliferation and infiltration into tumours, leading to tumour cell death [1]. In the European Union, ipilimumab 3 mg/kg is indicated for adult patients with advanced (unresectable or metastatic) melanoma. In randomised phase III trials, ipilimumab resulted in an overall survival (OS) benefit relative to control in both pretreated and treatment-naïve patients [2,3]. Additionally, long-term survival was observed with ipilimumab alone or in combination with other agents in phase II trials, with 5-year survival rates ranging from 13% to 36% [4-6].

Melanoma is associated with oncogenic mutations that activate the mitogen-activated protein kinase-signalling pathway, resulting in accelerated cell-cycle progression and enhanced proliferation [7]. Mutations in the genes encoding the protein kinases BRAF and NRAS are found in around 40% to 50%, and 20% of patients with cutaneous melanoma, respectively [8-10]. Agents approved to treat these subpopulations include the BRAF inhibitors vemurafenib and dabrafenib, and the MEK inhibitor trametinib, which are indicated for adult patients with BRAF^V600^ mutation-positive advanced melanoma [11-13]. Other inhibitors of mutated MEK and/or NRAS are in advanced clinical development [14]. However, responses to these agents may be short-lived due to the development of resistance and there is still a need to explore new therapeutic options [8].

Because ipilimumab targets the tumour indirectly, its mechanism of action is independent of kinase-signalling pathways. A retrospective analysis of data from a phase II trial showed that responses to ipilimumab were independent of BRAF^V600E^ mutation status, with 30% to 35% of all patients achieving disease control [15]. The immune-mediated mechanism of action results in immune-related adverse events (irAEs), caused by increased or excessive immune activity [16]. A relationship between irAE occurrence and response to ipilimumab was initially proposed following phase II data suggesting a link with longer survival [17-19]. However, these data were inconclusive, and disease control and survival benefits have also been observed in patients not experiencing irAEs [19,20].

Here we describe the safety and efficacy of ipilimumab 3 mg/kg in patients enrolled in an expanded access programme (EAP) in Italy. The EAP provided an opportunity to assess ipilimumab 3 mg/kg in a large cohort of advanced melanoma patients outside of a clinical trial setting (who had no other therapeutic options), with evaluation of the correlation between BRAF and NRAS mutation status and clinical benefit [21], and the potential relationship between efficacy and irAE development [22].

## Patients and methods

### Patients

Details of the EAP study in Italy are also reported elsewhere [23,24]. Adult patients, aged 16 or over, with unresectable stage III/IV cutaneous, ocular or mucosal melanoma were eligible if they had previously failed or were intolerant to ≥1 systemic therapy, had an Eastern Cooperative Oncology Group (ECOG) performance status (PS) of 0 to 2, and no other therapeutic option. Previous systemic treatment should have been completed ≥28 days before receiving ipilimumab. Previous treatment with an anti-CTLA-4 antibody was allowed unless prior use was discontinued for lack of clinical benefit or an AE. Patients with asymptomatic brain metastases at baseline were eligible.

### Study design and data collection

Patients received intravenous ipilimumab 3 mg/kg every 3 weeks for a total of four doses. In the absence of dose-limiting toxicities, patients with a stable PS received all four doses. Dose reduction or modification was not allowed, but dose omission or discontinuation was recommended when necessary. Patients with disease progression following stable disease (SD) of ≥3 months’ duration or an initial objective response (partial or complete) were eligible for retreatment with ipilimumab 3 mg/kg every 3 weeks for four doses.

The study has been conducted in accordance with all stipulations of the protocol and in accordance with Good Clinical Practices, local regulatory requirements (approved by a local Independent Ethics Committee) and the Declaration of Helsinki.

### Assessments

Tumour assessments were conducted at baseline and after completion of treatment (week 12) according to immune-related response criteria (irRC) [25]. Responses were classified as immune-related complete response (irCR), immune-related partial response (irPR), immune-related SD (irSD) or immune-related progressive disease (irPD). Clinical benefit was represented by immune-related best overall response rate (irBORR; irCR or irPR) and disease control rate (irDCR: percentage of patients achieving irCR, irPR or irSD). BRAF and NRAS mutation status was collected retrospectively, where possible.

AEs were monitored and assessed in all patients who received ipilimumab and graded according to the National Cancer Institute Common Terminology Criteria for Adverse Events (version 3). AEs were managed using protocol-specific guidelines. All AEs, regardless of drug attribution, were recorded from the first dose of ipilimumab until 70 days after treatment discontinuation.

### Statistical analysis

Patient and disease characteristics were analysed using descriptive statistics, and expressed as relative frequency (percentage) for discrete variables or median for continuous variables. Progression-free survival (PFS) and OS were estimated using Kaplan–Meier analysis and expressed as medians with corresponding two-sided 95% confidence intervals (CIs). Differences between curves were evaluated using the log-rank test.

## Results

### Patients

Between June 2010 and January 2012, 855 patients with cutaneous (*n* = 631), mucosal (*n* = 71), and ocular (*n* = 83) melanoma were treated with ipilimumab 3 mg/kg at one of 55 Italian centres in the EAP. Baseline characteristics are provided in Table 1. Most patients had an ECOG PS of 0 or 1, with only 25 patients (3%) having a PS of 2. At baseline, 17% had brain metastases and 40% had liver metastases. As per protocol, all patients had received prior systemic therapy, with 125 (15%) receiving three or more prior therapies. Although chemotherapy was the most frequently used previous treatment, 192 patients (22%) received prior immunotherapy with interferon and 59 patients (7%) had been treated with a BRAF inhibitor. Of 469 patients with tissue samples for retrospective testing of the BRAF mutation, 173 (37%) were positive. Fourteen of 82 patients (17%) tested for the NRAS mutation were positive, including three patients who also had a BRAF mutation.

**Table 1:** Baseline patient characteristics

**Characteristic**	***N*** **= 855**
Median age, years (range)	61 (16–88)
Male/female, *n* (%)	460 (54)/395 (46)
ECOG PS, *n* (%)	
0	563 (66)
1	267 (31)
2	25 (3)
Time from diagnosis, months (range)	39 (3–280)
Tumour subtype	
Cutaneous	631 (74)
Mucosal	71 (8)
Ocular	83 (10)
Primary origin unknown	70 (8)
Patients with brain metastases, *n* (%)	146 (17)
Patients with liver metastases, *n* (%)	339 (40)
Elevated LDH (≥1.10 ULN), *n*/*n* (%)	276/720 (38)
BRAF-mutation positive, *n*/*n* (%)	173/469 (37)
NRAS-mutation positive, *n*/*n* (%)	14/82 (17)
Number of previous therapies:	
1	497 (58)
2	233 (27)
≥3	125 (15)
Previous therapy, *n* (%)	
Dacarbazine	490 (57)
Fotemustine	322 (38)
Platinum-based chemotherapy	316 (37)
Paclitaxel	78 (9)
Temozolomide	189 (22)
Interferon	192 (22)
BRAF inhibitor	59 (7)

ECOG PS: Eastern Cooperative Oncology Group performance status; LDH: lactate dehydrogenase; ULN: upper limit of normal.

### Clinical response

With a median follow-up of 6.7 months (range, 0.5–34 months), the irBORR was 13%, comprising 29 patients (3%) with an irCR and 82 (10%) with an irPR at any time. Twenty-two patients were not evaluable for response for treatment refusal (*n =* 4), loss to follow-up (*n =* 7), treatment-related toxicity (*n = 3*), deterioration without progression (*n =* 3) or unknown reasons (*n =* 5). Of 833 evaluable patients, 175 (21%) had irSD, for an irDCR of 34%. The median duration of disease control was 13.1 months (95% CI, 11.1–15.0). Patients with BRAF-mutation positive tumours and BRAF wildtype tumours had comparable irDCRs (38% vs 39%), as did patients with or without NRAS mutations (57% vs 49%) (Table 2).

**Table 2:** Tumour response in all patients

	**Patients, *n *** **(%)**						
**Response according to irRC**	**Total (*N*** **= 833)**	**BRAF-mutation positive (*n*** **= 169)**	**BRAF wildtype (*n*** **= 291)**	**NRAS-mutation positive (*n*** **= 14)**	**NRAS wildtype (*n*** **= 67)**	**Any irAE (*n*** **= 278)**	**No irAE (*n*** **= 555)**
irCR	29 (3)	9 (6)	12 (4)	2 (14)	4 (6)	10 (4)	19 (3)
irPR	82 (10)	19 (11)	27 (9)	2 (14)	9 (13)	31 (11)	51 (9)
irSD	175 (21)	36 (21)	76 (26)	4 (29)	20 (30)	57 (21)	118 (21)
irPD	547 (66)	105 (62)	176 (61)	6 (43)	34 (51)	180 (65)	367 (66)
irDCR	286 (34)	64 (38)	115 (39)	8 (57)	33 (49)	98 (35)	188 (34)

irRC: immune-related response criteria; irAE: immune-related adverse event; irCR: immune-related complete response; irPR: immune-related partial response; irSD: immune-related stable disease; irPD: immune-related progressive disease; irDCR: immune-related disease control rate.

Of 855 treated patients, 51 (6%) were retreated with ipilimumab 3 mg/kg upon disease progression, including 12 patients with a BRAF mutation and two with an NRAS mutation. Of these 51 patients, the best response to induction therapy was an irCR or irPR in 20 patients and irSD lasting ≥3 months in 31 patients. Upon retreatment, the irDCR was 55%. Of the 12 patients with a BRAF mutation, two had an irCR, two had an irPR and four had irSD upon retreatment for an irDCR of 67%. Both NRAS-mutated patients had irPD upon retreatment.

**Figure 1:**
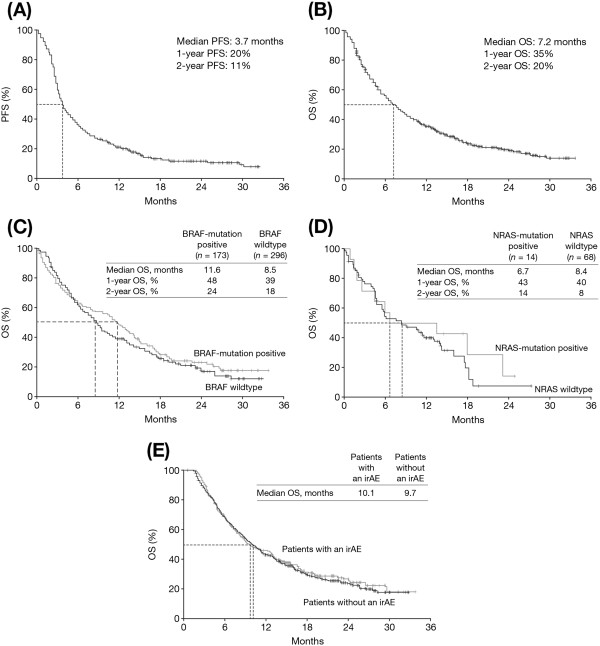
**Kaplan–Meier curves for PFS and OS.** PFS for all patients **(A)**. OS for all patients **(B)**. OS for BRAF-mutation positive (*n* = 173) and BRAF wildtype (*n* = 296) patients **(C)**. OS for NRAS-mutation positive (*n* = 14) and NRAS wildtype (*n* = 68) patients **(D)**. OS in patients with or without an irAE (any grade) in patients with who received three or four cycles of ipilimumab **(E)**. PFS: progression-free survival; OS: overall survival; irAE: immune-related adverse event.

As of December 2012, median PFS among all treated patients was 3.7 months (95% CI, 3.4–4.0; Figure 1A), and median OS was 7.2 months (95% CI, 6.4–8.0; Figure 1B). Survival curves were comparable between patients with and without mutations in BRAF or NRAS (Figure 1C) and Figure 1D). For patients with a BRAF mutation, median OS was 11.6 months (95% CI, 9.6–13.6) versus 8.5 months (95% CI, 7.4–9.6) for patients with wildtype BRAF. Similarly, median OS for patients with an NRAS mutation was 6.7 months (95% CI, 0–20.8) versus 8.4 months (95% CI, 3.9–12.8) for patients with wildtype NRAS. One and 2-year OS rates, as of December 2012, are presented in Figure 1.

### Safety and tolerability

Among all treated patients, 399 (47%) reported an AE (any grade), with grade 3/4 AEs in 100 patients (12%). AEs were generally reversible with treatment as per protocol-specific guidelines; 25 patients discontinued ipilimumab due to toxicity. AEs leading to discontinuation included diarrhoea (*n* = 7), rash (*n* = 2), liver dysfunction (*n* = 1), thrombocytopenia (*n* = 1), pain (*n* = 1) and vasculitis (*n* = 1). Five deaths were considered to be related to treatment (one patient each with tumour lysis syndrome, hypothermia, bone marrow aplasia, hepatitis and acute renal failure). For sites treating 1 to 10, 11 to 30, and over 30 patients, rates of hospitalisation for serious AEs were 9%, 5%, and 3%, respectively. The safety profile of ipilimumab was consistent regardless of BRAF and NRAS mutation status.

AEs were considered to be immune-related in 286 patients (33%) (Table 3), with a median time to onset of 5 weeks (range, 1–13 weeks).

**Table 3:** Summary of irAEs

**irAE**	**Patients, n (%)**	
	**Any grade**	**Grade 3/4**
Total	286 (33)	55 (6)
Pruritus	58 (7)	1 (<1)
Rash	64 (8)	4 (<1)
Diarrhoea	60 (7)	19 (2)
Nausea	47 (6)	2 (<1)
Vomiting	15 (2)	2 (<1)
Constipation	7 (1)	1 (<1)
Abdominal pain	11 (1)	0
Endocrine	7 (1)	1 (<1)
Liver toxicity	19 (2)	15 (2)
Fatigue/asthenia	70 (8)	10 (1)

irAEs: immune-related adverse events.

Baseline patient characteristics were similar for patients with or without irAEs. Most irAEs were low grade, with grade 3/4 irAEs in 55 patients (6%; most commonly diarrhoea, liver toxicity and fatigue/asthenia). Median time to resolution was 1.7 weeks (range, 0.1–11.1 weeks) for irAEs of any grade and 1.1 weeks (range, 0.1–3.4 weeks) for grade 3/4 irAEs.

Patients who were unable to complete at least three doses of ipilimumab (*n* = 194) had a lower incidence of irAEs (22%) than those who received three or more doses of ipilimumab (*n* = 661; 37% had irAEs), reflecting the onset of some irAEs beyond initial dosing.

### Relationship between efficacy and safety

The irDCRs for patients with or without an irAE of any grade were 35% and 34%, respectively (Table 2). Among the 278 patients with an irAE who were evaluable for response, 10 (4%) had an irCR, 31 (11%) an irPR and 57 (21%) irSD. For these patients, median duration of disease control was 11.7 months (95% CI, 9.9–13.5). Respective numbers for the 555 evaluable patients without an irAE were 19 (3%), 51 (9%) and 118 (21%), with a median duration of disease control of 13.6 months (95% CI, 11.1–6.0). After adjusting for the number of doses completed, median OS for patients who had an irAE was 10.1 months (95% CI, 8.9–11.3), compared with 9.7 months (95% CI, 7.1–12.3) for those who did not (Figure 1E).

## Discussion

Ipilimumab improved survival in phase III trials of both pretreated and treatment-naïve patients with metastatic melanoma [2,3]. To date, over 17,000 patients have been treated with ipilimumab, either commercially or through clinical trials or EAPs, establishing immune-oncology as one of the four pillars of anticancer treatment [26]. In the EAP, patients with no other therapeutic options, who were ineligible for clinical trials, received ipilimumab at its licensed dose, providing an opportunity to assess efficacy and safety in a large cohort of patients outside the trial setting. Disease control and survival data for more than 850 pretreated patients with metastatic melanoma were consistent with clinical trial data.

In the EAP in Italy, one third of patients achieved disease control at any time according to irRC, consistent with patients who received ipilimumab 3 mg/kg in the phase III registrational trial which used modified World Health Organization (WHO) criteria to evaluate bi-dimensionally measurable lesions [2]. Encouragingly, among 51 patients retreated with ipilimumab, 55% regained disease control, suggesting that restimulating an immune response may be an option for patients progressing after an initial response to treatment [27]. Responses and/or SD with ipilimumab can be durable, with PRs of 56 to 71+ months and CRs of 62 to 99+ months reported in phase II clinical trials [6]. Furthermore, clinical trial data consistently show a plateau in survival curves after 2 to 3 years, with approximately one fifth of patients having long-term survival, as confirmed with 10 years’ follow-up for some patients [4,5,28,29]. In preliminary landmark analyses from the EAP, an estimated 20% of patients were still alive at least 2 years after starting ipilimumab, suggesting these patients may also have additional life-expectancy. Extended follow-up would be required to confirm this.

Unlike vemurafenib, which is restricted to patients with the BRAF^V600^ mutation, ipilimumab can be used regardless of mutational status. In a previous study, DCRs were comparable between patients with BRAF^V600E^-mutated melanoma and those with wildtype tumours [15], and EAP data support this. In the EAP, both the irDCR and median OS appeared independent of BRAF and NRAS status. Ipilimumab, therefore, provides clinical benefit to patients with the BRAF^V600^ mutation, suggesting that optimal sequencing of ipilimumab and vemurafenib for BRAF-mutated metastatic melanoma should be investigated further as it may be beneficial to use ipilimumab earlier in the disease course.

Despite reproducible evidence of long-term clinical benefit with ipilimumab, some physicians are wary of the perceived severity and novelty of AEs resulting from increased or excessive immune activity. In the EAP in Italy, approximately one third of patients experienced an irAE of any grade, which were predominantly mild. A possible negative correlation between the number of patients treated at a site and the proportion experiencing serious AEs was observed, and AEs tended to resolve quicker than in clinical trials. In the registrational phase III trial of ipilimumab, median time to resolution of grade 3/4 immune-related AEs was 7.7 weeks (95% CI, 3.0–not reached) [2], compared with just 1.1 weeks (range, 0.1–3.4) in the EAP. These preliminary data suggest that as physicians gain more experience of treating patients with ipilimumab, they are more familiar with its associated AEs, enabling earlier detection and timely intervention. Recognition that managing AEs associated with ipilimumab is different, but no more difficult, than for AEs associated with non-immune agents may lead to increased confidence in ipilimumab use.

There appeared to be no obvious differences in the baseline characteristics of patients with and without an irAE. Because ipilimumab can break peripheral immune tolerance, there may be a relationship between irAEs and response to ipilimumab [1,18]. However, in the EAP, immune-related disease control and survival were comparable in patients with and without an irAE, suggesting that patients without an irAE of any grade can still benefit from ipilimumab as supported by previous data [19,20]. Continued vigilance and early intervention are recommended to address any symptoms and prevent potentially serious complications. Because irAEs can be severe or life-threatening, ipilimumab should be used with caution in patients with a history of autoimmune disease, and is contraindicated if the disease is active and severe.

This was an analysis of retrospective data from an EAP that was not designed to compare efficacy outcomes between patient subgroups, and used immune-related rather than WHO response criteria; therefore the results must be interpreted accordingly. Results from Italian centres may have also been influenced by national patient management trends. Nevertheless, the EAP provided an important opportunity to assess ipilimumab 3 mg/kg in a large cohort and a situation closely resembling daily clinical practice. Ipilimumab was generally well-tolerated, and efficacy did not appear related to irAE occurrence. The results suggest that ipilimumab is an effective and safe treatment for pretreated patients with metastatic melanoma regardless of BRAF and NRAS mutation status. Future research should focus on assessing the longer-term, real-life outcomes of ipilimumab therapy and increasing the number of patients who benefit through improved patient selection and timely intervention.

## Consent

All participating patients provided signed informed consent for administration of the drug, academic evaluation of clinical outcomes and publications or reports related to the compassionate use programme before enrolment. For patients below the legal age, consent from parents, guardians, or next of kin has been provided.

## Abbreviations

AEs: Adverse events; CIs: Confidence intervals; CTLA-4: Cytotoxic T-lymphocyte-associated antigen-4; EAP: Expanded access programme; ECOG PS: Eastern Cooperative Oncology Group performance status; irBORR: Immune-related best overall response rate; irCR: Immune-related complete response; irDCR: Immune-related disease control rate; irPD: Immune-related progressive disease; irPR: Immune-related partial response; irRC: Immune-related response criteria; irSD: Immune-related stable disease; OS: Overall survival; PFS: Progression-free survival; SD: Stable disease; WHO: World Health Organization.

## Competing interests

Paolo A. Ascierto has served in a consultancy/advisory role for Bristol-Myers Squibb (BMS), Merck Sharp & Dohme, Roche-Genentech, GlaxoSmithKline (GSK), Amgen and Celgene; he has also received research funding from BMS, and honoraria from BMS, Merck Sharp & Dohme, Roche-Genentech and GSK. Vanna Chiarion Sileni has served as a consultant and in an advisory role for BMS, GSK and Roche-Genentech, and has received support to attend meetings from BMS and GSK. Michele Maio has had an advisory role and received funding for communication programmes from BMS, Roche-Genentech and Merck Sharp & Dohme and has received research funding from BMS. Michele Del Vecchio has served as a consultant or in an advisory role for Roche-Genentech and GSK and has received research funding from Roche-Genentech, GSK, Celgene and BMS. Paolo Marchetti has served as a consultant or in an advisory role for BMS, GSK, Novartis, and Roche-Genentech. Alessandro Testori has served as a consultant or in an advisory role for, and received honoraria from, BMS, Roche-Genentech, GSK, Amgen, Merck Sharp & Dohme and IGEA, and travel expenses from IGEA and Oncovision. Paola Queirolo has served as a consultant and in an advisory role for BMS, GSK and Roche-Genentech. None of the remaining authors have any competing interests to disclose.

## Authors’ contributions

PAA, MM, VCS, and PQ devised the study concept. PAA, MM, and PQ were involved in designing the study and statistical analysis. All authors were responsible for the data acquisition, quality control of data and algorithms, and data analysis and interpretation. All authors contributed to the preparation, editing, and review of this manuscript. All authors read and approved the final manuscript.

## References

[B1] Hoos A Ibrahim R Korman A Abdallah K Berman D Shahabi V Chin K Canetta R Humphrey R Development of ipilimumab: contribution to a new paradigm for cancer immunotherapy Semin Oncol 2010 37 533 546 10.1053/j.seminoncol.2010.09.015 21074069

[B2] Hodi FS O'Day SJ McDermott DF Weber RW Sosman JA Haanen JB Gonzalez R Robert C Schadendorf D Hassel JC Akerley W van den Eertwegh AJ Lutzky J Lorigan P Vaubel JM Linette GP Hogg D Ottensmeier CH Lebbé C Peschel C Quirt I Clark JI Wolchok JD Weber JS Tian J Yellin MJ Nichol GM Hoos A Urba WJ Improved survival with ipilimumab in patients with metastatic melanoma N Engl J Med 2010 363 711 723 10.1056/NEJMoa1003466 20525992 PMC3549297

[B3] Robert C Thomas L Bondarenko I O'Day S JW MD Garbe C Lebbe C Baurain JF Testori A Grob JJ Davidson N Richards J Maio M Hauschild A Miller WH Jr Gascon P Lotem M Harmankaya K Ibrahim R Francis S Chen TT Humphrey R Hoos A Wolchok JD Ipilimumab plus dacarbazine for previously untreated metastatic melanoma N Engl J Med 2011 364 2517 2526 10.1056/NEJMoa1104621 21639810

[B4] Lebbé C Weber JS Maio M Neyns B Harmankaya K Hamid O O'Day S Chin KM Opatt McDowell D Cykowski L McHenry B Wolchok JD Long-term survival in patients with metastatic melanoma who received ipilimumab in four phase II trials [abstract] J Clin Oncol 2013 31 suppl 9053

[B5] Wolchok JD Weber JS Maio M Neyns B Harmankaya K Chin K Cykowski L de Pril V Humphrey R Lebbé C Four-year survival rates for patients with metastatic melanoma who received ipilimumab in phase II clinical trials Ann Oncol 2013 24 2174 2180 10.1093/annonc/mdt161 23666915 PMC4081656

[B6] Prieto PA Yang JC Sherry RM Hughes MS Kammula US White DE Levy CL Rosenberg SA Phan GQ CTLA-4 blockade with ipilimumab: long-term follow-up of 177 patients with metastatic melanoma Clin Cancer Res 2012 18 2039 2047 10.1158/1078-0432.CCR-11-1823 22271879 PMC3319861

[B7] Arkenau HT Kefford R Long GV Targeting BRAF for patients with melanoma Br J Cancer 2011 104 392 398 10.1038/sj.bjc.6606030 21139585 PMC3049553

[B8] Sullivan RJ Flaherty KT Resistance to BRAF-targeted therapy in melanoma Eur J Cancer 2013 49 1297 1304 10.1016/j.ejca.2012.11.019 23290787

[B9] Long GV Menzies AM Nagrial AM Haydu LE Hamilton AL Mann GJ Hughes TM Thompson JF Scolyer RA Kefford RF Prognostic and clinicopathologic associations of oncogenic BRAF in metastatic melanoma J Clin Oncol 2011 29 1239 1246 10.1200/JCO.2010.32.4327 21343559

[B10] Spagnolo F Queirolo P Upcoming strategies for the treatment of metastatic melanoma Arch Dermatol Res 2012 304 177 184 10.1007/s00403-012-1223-7 22350184

[B11] Chapman PB Hauschild A Robert C Larkin J Haanen J Ribas A Hogg D Hamid O Ascierto PA Testori A Lorigan P Dummer R Sosman JA Garbe C Maio M Nolop KB Nelson BJ Joe AK Flaherty KT McArthur GA Updated overall survival (OS) results for BRIM-3, a phase III randomized, open-label, multicenter trial comparing BRAF inhibitor vemurafenib (vem) with dacarbazine (DTIC) in previously untreated patients with BRAFV600E-mutated melanoma [abstract] J Clin Oncol 2012 30 suppl 15 8502

[B12] Hauschild A Grob JJ Demidov LV Jouary T Gutzmer R Millward M Rutkowski P Blank CU Miller WH Kaempgen E Martin-Algarra S Karaszewska B Mauch C Chiarion-Sileni V Mirakhur B Guckert ME Swann RS Haney P Goodman VL Chapman PB An update on BREAK-3, a phase III, randomized trial: dabrafenib versus dacarbazine in patients with BRAF V600E-positive mutation metastatic melanoma [abstract] J Clin Oncol 2013 31 suppl 9013

[B13] Flaherty KT Robert C Hersey P Nathan P Garbe C Milhem M Demidov LV Hassel JC Rutkowski P Mohr P Dummer R Trefzer U Larkin JM Utikal J Dreno B Nyakas M Middleton MR Becker JC Casey M Sherman LJ Wu FS Ouellet D Martin AM Patel K Schadendorf D METRIC Study Group Improved survival with MEK inhibition in BRAF-mutated melanoma N Engl J Med 2012 367 107 114 10.1056/NEJMoa1203421 22663011

[B14] Ascierto PA Schadendorf D Berking C Agarwala SS van Herpen CM Queirolo P Blank CU Hauschild A Beck JT St-Pierre A Niazi F Wandel S Peters M Zubel A Dummer R MEK162 for patients with advanced melanoma harbouring NRAS or Val600 BRAF mutations: a non-randomised, open-label phase 2 study Lancet Oncol 2013 14 249 256 10.1016/S1470-2045(13)70024-X 23414587

[B15] Shahabi V Whitney G Hamid O Schmidt H Chasalow SD Alaparthy S Jackson JR Assessment of association between BRAF-V600E mutation status in melanomas and clinical response to ipilimumab Cancer Immunol Immunother 2012 61 733 737 10.1007/s00262-012-1227-3 22382362 PMC11029315

[B16] Weber JS Kähler KC Hauschild A Management of immune-related adverse events and kinetics of response with ipilimumab J Clin Oncol 2012 30 2691 2697 10.1200/JCO.2012.41.6750 22614989

[B17] Attia P Phan GQ Maker AV Robinson MR Quezado MM Yang JC Sherry RM Topalian SL Kammula US Royal RE Restifo NP Haworth LR Levy C Mavroukakis SA Nichol G Yellin MJ Rosenberg SA Autoimmunity correlates with tumour regression in patients with metastatic melanoma treated with anti-cytotoxic T-lymohocyte antigen-4 J Clin Oncol 2005 23 6043 6053 10.1200/JCO.2005.06.205 16087944 PMC1473965

[B18] Downey SG Klapper JA Smith FO Yang JC Sherry RM Royal RE Kammula US Hughes MS Allen TE Levy CL Yellin M Nichol G White DE Steinberg SM Rosenberg SA Prognostic factors related to clinical response in patients with metastatic melanoma treated by CTL-associated antigen-4 blockade Clin Cancer Res 2007 13 6681 6688 10.1158/1078-0432.CCR-07-0187 17982122 PMC2147083

[B19] Tarhini A Lo E Minor DR Releasing the brake on the immune system: ipilimumab in melanoma and other tumours Cancer Biother Radiopharm 2010 25 601 613 10.1089/cbr.2010.0865 21204754 PMC3011989

[B20] Lutzky J Wolchok J Hamid O Lebbe C Pehamberger H Linette G de Pril V Ibrahim R Hoos A O'Day S Ipilimumab exerts disease control and survival benefits in advanced melanoma patients with and without immune-related adverse events [abstract] J Clin Oncol 2009 27 suppl 15 9034

[B21] Queirolo P Spagnolo F Altomonte M Chiarion-Sileni V Pigozzo J Del Vecchio M Di Guardo L Ridolfi R Scoppola A Ferrucci PF Ferraresi V Bernengo MG Guida M Marconcini R Mandalà M Parmiani G Rinaldi G Aglietta M Simeone E Ascierto PA Italian cohort of the ipilimumab Expanded Access Programme: efficacy, safety and correlation with mutation status in metastatic melanoma patients [abstract] J Clin Oncol 2013 31 suppl 9070

[B22] Di Giacomo AM Grimaldi AM Ascierto PA Queirolo P Del Vecchio M Ridolfi R De Rosa F De Galitiis F Testori A Cognetti F Bernengo MG Savoia P Guida M Strippoli S Galli L Mandala M Parmiani G Rinaldi G Aglietta M Chiarion-Sileni V Correlation between efficacy and toxicity in patients with pretreated advanced melanoma treated with the Italian cohort of the ipilimumab Expanded Access Programme [abstract] J Clin Oncol 2013 31 suppl 9065

[B23] Del Vecchio M Di Guardo L Ascierto PA Grimaldi AM Sileni VC Pigozzo J Ferraresi V Nuzzo C Rinaldi G Testori A Ferrucci PF Marchetti P De Galitiis F Queirolo P Tornari E Marconcini R Calabrò L Maio M Efficacy and safety of ipilimumab 3 mg/kg in patients with pretreated, metastatic, mucosal melanoma Eur J Cancer 2014 50 121 127 10.1016/j.ejca.2013.09.007 24100024

[B24] Maio M Danielli R Chiarion-Sileni V Pigozzo J Parmiani G Ridolfi R De Rosa F Del Vecchio M Di Guardo L Queirolo P Picasso V Marchetti P De Galitiis F Mandalà M Guida M Simeone E Ascierto PA Efficacy and safety of ipilimumab in patients with pre-treated, uveal melanoma Ann Oncol 2013 24 2911 2915 10.1093/annonc/mdt376 24067719

[B25] Wolchok JD Hoos A O'Day S Weber JS Hamid O Lebbé C Maio M Binder M Bohnsack O Nichol G Humphrey R Hodi FS Guidelines for the evaluation of immune therapy activity in solid tumours: immune-related response criteria Clin Cancer Res 2009 15 7412 7420 10.1158/1078-0432.CCR-09-1624 19934295

[B26] Wolchok JD Hodi FS Weber JS Allison JP Urba WJ Robert C O'Day SJ Hoos A Humphrey R Berman DM Lonberg N Korman AJ Development of ipilimumab: a novel immunotherapeutic approach for the treatment of advanced melanoma Ann NY Acad Sci 2013 1291 1 13 10.1111/nyas.12180 23772560 PMC3910157

[B27] Pigozzo J Ascierto PA Calabrò L De Galitiis F Ferrucci PF Queirolo P Bernengo MG Aglietta M Mandala M Del Vecchio M Efficacy and safety of ipilimumab reinduction therapy in patients with pretreated advanced melanoma participating in an Expanded Access Programme in Italy [abstract] Ann Oncol 2012 23 suppl 9 1131P

[B28] Maio M Bondarenko I Robert C Thomas L Garbe C Testori A Francis S Chin K Wolchok J Four-year survival update for metastatic melanoma patients treated with ipilimumab plus dacarbazine in phase 3 study CA184-024 [abstract] Ann Oncol 2012 23 suppl 9 1127P

[B29] Schadendorf D Hodi FS Robert C Weber JS Margolin K Hamid O Chen TT Berman DM Wolchok JD Pooled analysis of long-term survival data from Phase II and Phase III trials of ipilimumab in metastatic or locally advanced, unresectable melanoma [abstract] Eur J Cancer 2013 49 suppl 24LBA 10.1200/JCO.2014.56.2736 PMC5089162 25667295

